# Deficits in the Proline-Rich Synapse-Associated Shank3 Protein in Multiple Neuropsychiatric Disorders

**DOI:** 10.3389/fneur.2017.00670

**Published:** 2017-12-11

**Authors:** Peter N. Alexandrov, Yuhai Zhao, Vivian Jaber, Lin Cong, Walter J. Lukiw

**Affiliations:** ^1^Russian Academy of Medical Sciences, Moscow, Russia; ^2^LSU Neuroscience Center, Louisiana State University Health Sciences Center New Orleans, New Orleans, LA, United States; ^3^Department of Anatomy and Cell Biology, Louisiana State University Health Sciences Center, New Orleans, LA, United States; ^4^Department of Neurology, Shengjing Hospital, China Medical University, Shenyang, China; ^5^Department of Ophthalmology, Louisiana State University Health Sciences Center New Orleans, New Orleans, LA, United States; ^6^Department of Neurology, Louisiana State University Health Sciences Center New Orleans, New Orleans, LA, United States

**Keywords:** 5x familial Alzheimer’s disease transgenic mice, 42 amino acid amyloid-beta peptides, aluminum sulfate, Alzheimer’s disease, autism spectrum disorder, bipolar disorder-schizophrenia, neurotransmission, Shank3 protein

## Abstract

Signaling between neurons in the human central nervous system (CNS) is accomplished through a highly interconnected network of presynaptic and postsynaptic elements essential in the conveyance of electrical and neurochemical information. One recently characterized core postsynaptic element essential to the efficient operation of this complex network is a relatively abundant ~184.7 kDa proline-rich synapse-associated cytoskeletal protein known as Shank3 (SH3-ankyrin repeat domain; encoded at human chr 22q13.33). In this “*Perspectives*” article, we review and comment on current advances in Shank3 research and include some original data that show common Shank3 deficits in a number of seemingly unrelated human neurological disorders that include sporadic Alzheimer’s disease (AD), autism spectrum disorder (ASD), bipolar disorder (BD), Phelan–McDermid syndrome (PMS; 22q13.3 deletion syndrome), and schizophrenia (SZ). Shank3 was also found to be downregulated in the CNS of the transgenic AD (TgAD) 5x familial Alzheimer’s disease murine model engineered to overexpress the 42 amino acid amyloid-beta (Aβ42) peptide. Interestingly, the application of known pro-inflammatory stressors, such as the Aβ42 peptide and the metal-neurotoxin aluminum sulfate, to human neuronal–glial cells in primary culture resulted in a significant decrease in the expression of Shank3. These data indicate that deficits in Shank3-expression may be one common denominator linking a wide-range of human neurological disorders that exhibit a progressive or developmental synaptic disorganization that is temporally associated with cognitive decline.

## Shank3—An Essential Postsynaptic Scaffolding Protein

A small gene family of proline-enriched synapse-associated “*SH3 and multiple ankyrin repeat domain*” proteins known as Shank1, Shank2, and Shank3 encode abundant postsynaptic scaffolding proteins highly enriched at glutamatergic synapses in the human and murine central nervous system (CNS) (1–5). All three Shank genes have alternative promoter options and complex intron/exon arrangements resulting in the generation of a complex array of mRNA transcripts and protein isoforms. For example, Shank3 (also known as ProSAP2), the best studied of the three Shank proteins, is encoded at mouse chromosome 15E3 (analogous to human chr 22q13.3), spans ~60 kb of genomic DNA, has 22 exons and multiple intragenic promoters and several alternative splicing exons, and is highly expressed in CNS neurons (1, 2, 4, 6). As part of this small Shank gene family, the neuronal-enriched, multi-domain integral scaffolding protein Shank3 functions: (i) to organize and interconnect multiple postsynaptic-membrane proteins, ionic-channel and neurotransmitter receptors to the β-actin-enriched microfilament cytoskeletal-system; (ii) to regulate synaptic development, function, and plasticity by orchestrating the assembly of postsynaptic-signaling complexes, maintaining dendritic spine and synaptic architecture and supporting G-protein-coupled signaling-pathways (1–3, 5, 7). To add to this complexity, Shank3 gene expression appears to be regulated by epigenetic mechanisms that include DNA methylation, histone acetylation, and posttranscriptional regulators involving microRNA activities, thus contributing to the temporal and tissue-specific expression of different Shank3 isoforms (8, 9). Different Shank3 protein isoforms are alternately expressed according to brain region, cell type, and developmental stage; for example, (i) all five major Shank3 isoforms (Shank 3A-3E) are highly abundant in mouse hippocampal neurons throughout development and aging (1–3, 7); (ii) full length human Shank3 (1,731 amino acids; 184,667 Da) contains six highly interactive domains in tandem conducive to engagement in multiple protein–protein interactions at the postsynaptic density (PSD) (1–4); (iii) posttranscriptional regulation of Shank3 expression may be mediated by microRNAs, such as miRNA-34a, that itself has been implicated in multiple neuropsychiatric disorders involving synaptic disruption (8–11); and (iv) Shank3 possesses a remarkably complex interactome, conducive to Shank3’s role as a master organizer of a highly interconnected synaptic and cytoskeletal network (12–17). This involves the 4–7 nm diameter β-actin microfilaments, the major cytoskeletal protein found in the PSD, and the 10 nm diameter intermediate filaments within the soma, neurites, and synapses of neuronal cells (1–4, 6, 11, 14, 18, 19).

## Alzheimer’s Disease (AD), Autism Spectrum Disorder (ASD), Bipolar Disorder (BD), Phelan–McDermid Syndrome (PMS), and Schizophrenia (SZ)—Shank3-Mediated Synaptic Degeneration and Cognitive Disability

**Figure 1:**
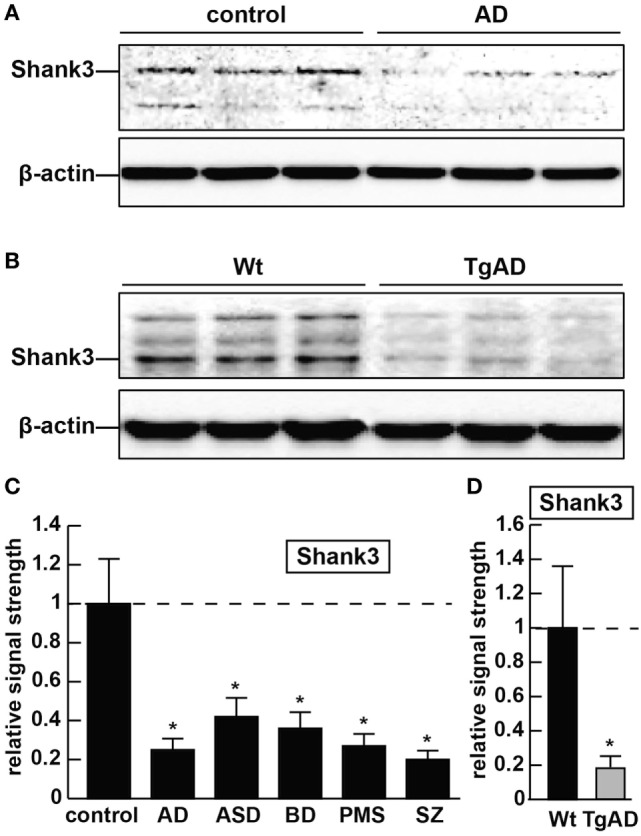
The abundance of the ~184.7 kDa Shank3 protein is decreased in synaptosome preparations from multiple human neurological disorders; **(A)** Shank3 protein levels are reduced in age- and gender-matched (all female) human brain temporal lobe neocortex from sporadic Alzheimer’s disease (AD) patients (control and AD; upper panel) when compared with control β-actin signals in the same sample [mean age ± 1 SD; control mean age 75 ± 8.3 years, *N* = 6; AD mean age 77 ± 6.5 years, *N* = 3]; all postmortem intervals (PMI; interval of death-to brain-freezing at −81°C) were 3 h or less; β-actin antibody 3598-100; Sigma-Aldrich, St. Louis, MO, USA and human Shank3 monoclonal antibody (C-4; sc-10479: H-75; sc-377088; Santa Cruz Biotechnologies, Santa Cruz, CA, USA; the main band at ~184.7 kDa is Shank3 protein); **(B)** similarly the levels of Shank3 protein are reduced in murine brain cortex from 7-month-old 5x familial Alzheimer’s disease (5xFAD) transgenic animals versus wild-type C57BL/6 age-matched controls; wild-type (Wt) and TgAD (5xFAD) murine models compared with β-actin signals in the same sample, as demonstrated by representative Western blot analysis using methods previously described in detail by our laboratory and as suggested by the manufacturer (11, 36, 37); modulation of actin dynamics at the synapse is likely to drive the cytoarchitectural changes that are associated with synaptic plasticity; in panels **(A,B)** multiple bands for Shank3 protein on Western gels may be indicative of alternate translation products from differentially spliced Shank3 mRNAs and/or amino acid side-chain modification [(1–5, 7); see text]; **(C)** bar graphs representative of Shank3 protein levels in age- and gender-matched (all female) human brain temporal lobe neocortex in control, AD, autism spectrum disorder (ASD), bipolar disorder (BD), Phelan–McDermid syndrome (PMS) (22q13.3 deletion syndrome), and schizophrenia (SZ) using Western blot analysis of synaptosome-enriched fractions [prepared using differential gradient centrifugation (12, 38–40)]; for control and AD, mean age ± 1 SD is given above; for all six tissue types PMIs were 3 h or less; ASD, BD, PMS, or SZ cases each had their own individual age-matched controls set to 1.0 in panel **(C)**; there were no significant differences in age between ASD, BD, PMS, or SZ cases and their individual controls; mean ages for ASD, BD, PMS, and SZ cases were 7.2 ± 2.7, 41.1 ± 7.1, 38.4 ± 6.8, and 44.3 ± 6.5 years, respectively; in these neurological disorders, Shank3 protein abundance was observed to be reduced from 0.21-fold (SZ) to 0.42-fold (ASD) of controls; **(D)** Shank3 protein abundance is similarly reduced to approximately 0.2-fold of control in the cortex of 7-month-old 5xFAD TgAD models; *N* = 3–6 samples of each neurological deficit or control; a dashed horizontal line at 1.0 has been included for ease of comparison; bars are the mean ± 1 SD of that mean; *p < 0.01 (ANOVA).

**Figure 2:**
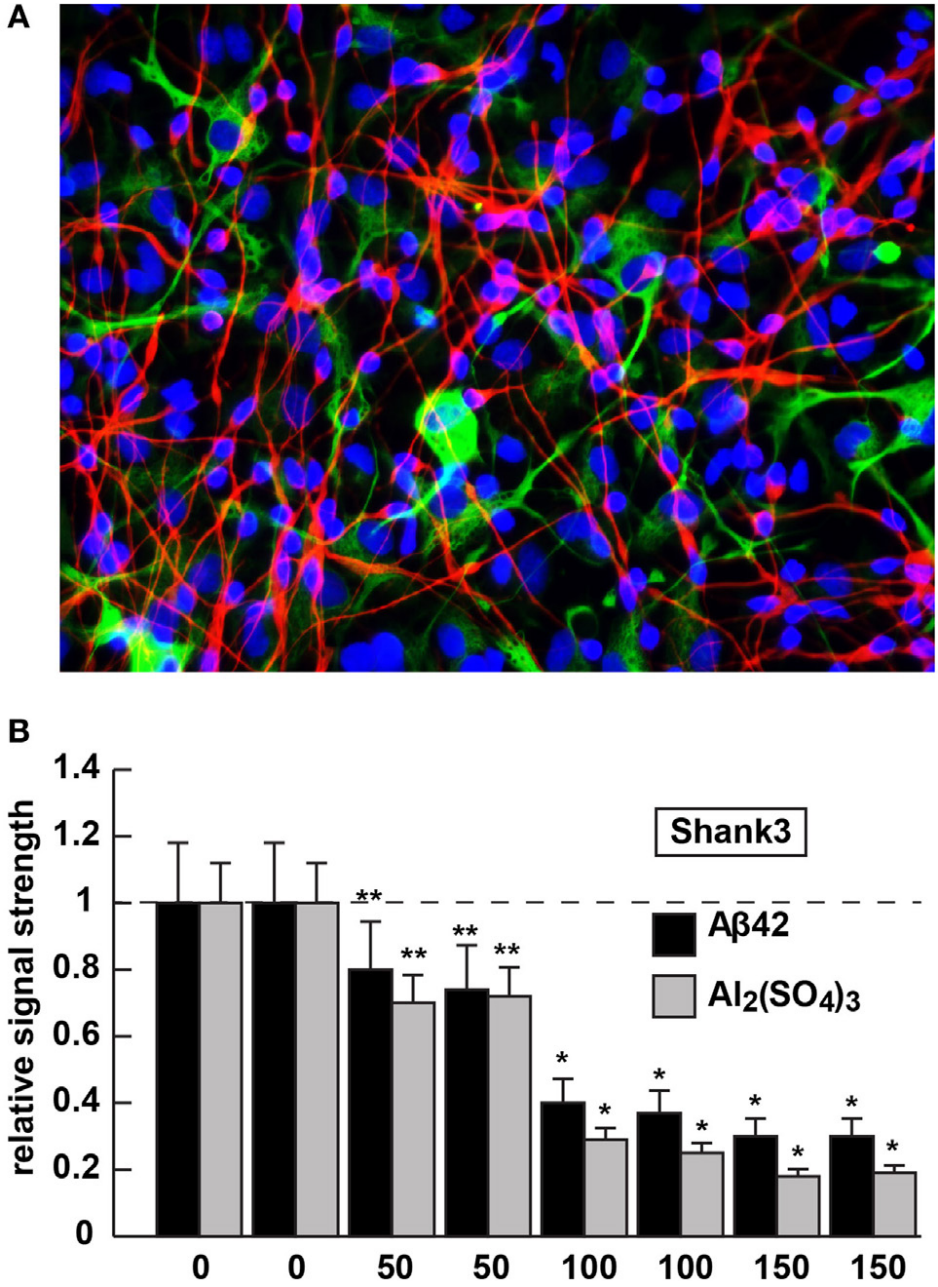
Shank3 downregulation in 42 amino acid amyloid-beta (Aβ42) peptide- or aluminum sulfate [Al_2_(SO_4_)_3_]-stressed human neuronal–glial (HNG) cells in primary coculture; **(A)** 2-week-old HNG cocultures (approximately 60% neurons and 40% astroglia) stained with the neuron-specific marker β tubulin 3 (red signal; λ_max_ ~680 nm) or the astroglial-specific marker GFAP (green signal; λ_max_ ~550 nm); HNG nuclei have been also stained with DAPI (blue; λ_max_ ~430 nm); culture of HNG cells has been previously described in detail by our laboratory (19, 41, 42); human brain neurons do not culture well without the presence of astroglial cells; magnification 40×; HNG cocultures were incubated with Aβ42 peptide (0, 50, 100, or 150 nM for 36 h) or ultrapure aluminum sulfate [Al_2_(SO_4_)_3_; 0, 50, 100 or 150 nM for 36 h]; these concentrations of stressors and times were selected from previous reports of Aβ42 peptide- and aluminum sulfate-induced inflammatory neurodegeneration and other relevant reports on neurotoxicity toward HNG cells, human brain microvessel endothelial cells that line the cerebral vasculature and other brain cell types (25–27, 36, 42–47); methodologies involving the application of Aβ42 peptide- or aluminum sulfate as physiologically realistic stressors to HNG cells in primary coculture have been explained in detail in previously published reports from our laboratory (11, 36, 42, 45–47); HNG whole-cell protein extracts were prepared, and Shank3 abundance was quantified using Western analysis and ImageQuant as described in Figures 1A,B (10, 36, 37, 43); **(B)** results are quantified in bar-graph format; at 100 nM Aβ42 peptide- or aluminum sulfate-treatment Shank3 levels were reduced between 0.2- and 0.3-fold of untreated control values; *N* = 3–5 samples of each treatment or condition; in Figure 2B, a dashed horizontal line at 1.0 has been included for ease of comparison; bars represent the mean ± 1 SD; *p < 0.01; **p < 0.05 (ANOVA).

As forementioned alternate Shank3 protein isoforms are differentially expressed according to developmental stage, cell type, and course of aging, suggesting the existence of isoform-specific roles for Shank3 at varying subcellular localizations, along different CNS neurites and synaptic endings at different stages of development and disease. Shank3 mutations that include gene breakpoints, deletions, point mutations, missense mutations, microdeletions, and nonsense mutations are associated with moderate to severe intellectual disabilities (2–7). AD with an incidence of one in seven people at age of 65–74 years and one in three people at age of 85 years and older, and the most common cause of intellectual decline associated with aging, is characterized by neuronal cell atrophy, swelling of neuronal nuclei, synaptic atrophy and loss, inflammatory neurodegeneration, progressive memory impairment, and a devastating cognitive decline (20–24). In AD brain tissues, in parallel, occurs the appearance of the 42 amino acid amyloid-beta (Aβ42) peptide-containing extracellular lesions, and although controversial, the accumulation of metallic neurotoxins such as aluminum (23–27). Shank3 was found to be reduced to ~0.25-fold of controls in AD neocortex (Figure 1). Shank3 gene mutations were first implicated (i) in ASD, a developmental disorder involving the disruption of social skills, speech and nonverbal communication, repetitive behaviors, and cognitive disabilities characterized pathologically by disordered gray and white matter, consistent changes in the densities of dendritic spines, region-specific abnormalities in neuronal morphology and cytoarchitectural organization, impairment in synaptic plasticity and progressive synaptic disorganization (28–30); (ii) in PMS, a developmental disorder that is caused by a 22q13.3 deletion and is characterized by ASD-like behavior, hypotonia, delayed or absent speech, intellectual disability, and cognitive impairment (28, 29, 31). Interestingly, patients with Shank3 mutations appear to have more-severe cognitive deficits than those with Shank1 or Shank2 mutations, and suggest that Shank3 mutation screening in clinical practice, and perhaps the restoration of the Shank3 gene activity may selectively rescue pathogenic synaptic defects of some ASD-associated behavioral phenotypes (7, 28, 29). Similar attributes in BD and SZ involving malformations in neuronal morphology, abnormalities in cytoarchitectural organization, impairment in synaptic plasticity and synaptic disorganization, impairment or insufficiency in a large portfolio of synaptic adhesion/organizing molecules, and cognitive disability are widely reported, and three extremely comprehensive studies have recently appeared (32–34). Also very recently, genetic- and bioinformatics-based analysis of brain region-specific Shank3 interactomes has been generated, which may be useful for understanding the heterogeneity of neuronal pathophysiology related to Shank3 genetic mutations and alternate Shank3 isoforms (12–15, 33–35). Here, we report the common downregulation in the expression of Shank3 in sporadic AD, ASD, BD, PMS, and SZ in 7-month-old 5x familial Alzheimer’s disease (5xFAD) transgenic animals exhibiting extensive amyloid deposition, compared with age-matched controls and in stressed human neuronal–glial (HNG) cell cocultures (see below; Figures 1 and 2). Ongoing work is currently in progress to further compare other neurological diseases involving progressive synaptic disorganization and intellectual disability, including attention deficit hyperactivity disorder, epilepsy, prion disease and others for Shank3 deficits, and how different “*loss-of-function*” mutations or altered Shank3 expression can lead to such phenotypic diversity.

## Aβ42 Peptide or Aluminum Sulfate [Al_2_(SO_4_)_3_] Downregulate Shank3 Expression

The 42 amino acid amyloid-beta peptide and aluminum (sulfate) were chosen as highly relevant pathogenic neurological stressors for the experimental treatment of HNG cells and assessment of Shank3 expression for the following reasons (see also Figure 2). First, the highly amyloidogenic Aβ42 peptides are one of the two key molecular lesions associated with AD (the other being and hyperphosphorylated tau), and multiple transgenic murine models for AD (TgAD) and involving massive amyloid overexpression have been generated (23, 24). The 5xFAD murine TgAD model containing five familial AD mutations; including three amyloid mutations and two presenilin 1 (PSEN1) mutations [APP KM670/671NL (Swedish), APP I716V (Florida), APP V717I (London), PSEN1 M146L (A>C), and PSEN1 L286V] exhibit substantial Aβ42 peptide generation, amyloid plaque deposition, astrogliosis, and cognitive impairment by 7 months of age and also exhibit deficits in Shank3 at this time point [(25); Figure 1].

Aluminum is a ubiquitous metallic neurotoxin and extremely potent genotoxin (25–27). Human intake of an average of 10 mg Al/day (range 10–1,000 mg Al/day) occurs chiefly *via* the ingestion of drinking water, food, medicine, and inhalation of airborne dust (43, 44, 48). Fortunately, the low solubility of aluminum at biological pH and highly evolved endothelial- and epithelial cell-based gastrointestinal and blood–brain barriers prevent this potent and ubiquitous metallotoxin from easy access to human biological compartments. However, aluminum that does gain entry into the CNS in animal models has been shown to induce NF-κB and specific microRNA-mediated inflammatory and neuro-immune pathogenic gene expression programs that closely emulate many aspects of CNS pathology and progressive memory dysfunction, including synaptic disorganization, as seen in advanced late stage AD brain (43, 44, 48). Our laboratory and others, for example, have further shown (i) the aluminum-mediated downregulation of several key brain essential genes in multiple neurodegenerative disorders by a pro-inflammatory NF-κB-regulated microRNA-146a (27, 36, 43, 45, 46, 48); (ii) a robust upregulation of microRNA-34a expression by reactive oxygen species and NF-κB increases in the presence of aluminum sulfate in HNG cocultures that decreases the expression of Shank3 [see Figure 2; (36, 46)]. We speculate that this may contribute to altered neurotransmission in multiple neuropsychiatric disorders [(25–27); unpublished observations]. The Shank3 downregulation in Aβ42 peptide- or aluminum sulfate-stressed HNG cells in primary culture (at concentrations of 0, 50, 100, or 150 nM for 36 h) is shown in Figure 2. Altered neurotransmission and synaptic dysfunction in the presence of amyloid peptides or neurotoxic metal salts are widely documented in very recent reviews in this subject area (25–27, 31, 41, 43, 44, 48–50).

## Concluding Remarks

Multifactorial diseases that include sporadic AD, ASD, BD, PMS, and SZ exhibit considerable heterogeneity in their presentation, and on the surface appear to exhibit more neuropathological variability than commonality. However, current research has advanced our understanding of the dynamics of the postsynaptic multi-domain proline-rich synapse-associated Shank3 protein and, perhaps surprisingly, has revealed a common Shank3-mediated “generic” disruption of synaptic organization in each of these neurological disorders. Interestingly, in neurological diseases involving synaptic disruption and cognitive decline, not all synaptic proteins appear to be equally affected. For example, the PSD-, β-actin-, and Shank3-associated scaffolding protein Homer 1 is decreased in AD but not in SZ brains [unpublished observations; (35)], and the relatively abundant PSD-95-associated DLGAP scaffold protein subtype DLGAP4 is significantly decreased in SZ but not in BD (15). Recently published studies including our own provides at least five novel and significant findings: (i) that the essential scaffolding protein Shank3 appears to be commonly and significantly reduced in synaptosomal preparations of brain tissues obtained from AD, ASD, BD, PMS, and SZ patients (2, 6–8, 37); (ii) that in the 5xFAD amyloid-overexpressing TgAD model [bearing the APP KM670/671NL (Swedish), APP I716V (Florida), APP V717I (London), PSEN1 M146L (A>C), and PSEN1 L286V mutations], the accumulation of Aβ42 peptides in the brain appears to be accompanied by reduced bioavailability of Shank3 (Figures 1 and 2); (iii) that research implicating Shank3 as an essential postsynaptic cytoskeletal organizing protein infers that the presence of Aβ42 peptides driving Shank3 downregulation would also be disruptive toward normal synaptic structure and signaling capabilities (12–17); (iv) that deficits in Shank3 abundance can be induced in HNG cells in primary coculture from the peripheral application of AD-relevant stressor substances, at physiologically realistic concentrations, that include Aβ42 peptides or environmentally abundant neurotoxins such as aluminum sulfate (42–44, 48); and (v) that Aβ42 peptide, aluminum sulfate, and perhaps other related intrinsic or environmental neurotoxins *via* depletion of essential synaptic proteins such as Shank3 can be detrimental to the homeostatic maintenance of synaptic structure, function, and plasticity, and may be conducive to a phenotype involving progressive cognitive insufficiency (42–44, 47, 48, 51).

Importantly, these experimental interpretations should remain be somewhat speculative as they are derived from preliminary studies, and more research is required to further clarify the significance of Shank3 deficits in other neurological disorders and experimental models. Whether Shank3 downregulation is a direct cause or consequence of these neurological disorders, or an unrelated epiphenomenon remains open to question. However, several recent studies of Shank3’s extensive and remarkably diverse interactome further underscore the idea that this highly interconnected synaptic protein is a “*master cytoskeletal hub*” within the PSD network. This further suggests that Shank3 disruption or deficiency could contribute to multiple neurological disorders associated with: (i) altered neurogenesis and disrupted expression of synaptic proteins that affect synaptic organization and plasticity; (ii) altered activation of cell adhesion molecules such as integrin; (iii) developmental and morphogenetic abnormalities; (iv) changes in biometal abundances and transporters such as those for zinc; (v) alterations in G protein-coupled metabotropic glutamate receptor 5 (mGluR5)-Homer scaffolds and mGlu5 receptors; and (vi) peripheral aberrations such as abnormally heightened sensitivity to pain in diseases associated with Shank3 deficits or in Shank3 animal models (12–17, 47, 51–61). From these most recent findings, we may further speculate (i) that deficits in Shank3 expression and synaptic structural disorganization represent a common underlying mechanism for neurological disorders, which exhibit abnormal synaptic dynamics and a neurodegenerative phenotype; (ii) predict that detectable alterations in Shank3 gene structure and/or alternate Shank3 mRNA isoforms screened *in utero* or in newborns may be diagnostic for the onset of neurological disorders such as AD, ASD, BD, PMS, and SZ in later life; and (iii) propose that pharmacological approaches including anti-microRNA strategies directed toward the manipulation of Shank3 and/or related synaptic protein expression may be useful in the clinical management of multiple neuropsychiatric disorders, which exhibit an underlying commonality in intellectual disability and/or progressive cognitive decline.

## Ethics Statement

All procedures involving murine and postmortem human tissues were followed and handled in strict accordance with the ethics review board policies at donor institutions and the Institutional Biosafety Committee/Institutional Review Board (IBC/IRB) ethical guidelines at the LSU Health Sciences Center, LA 70112 (IBC# 12323; IRB# 6774).

## Author Contributions

PA, LC, VJ, and YZ performed the experiments; YZ and WL analyzed the data; WL organized the data and wrote the article.

## Conflict of Interest Statement

The authors declare that the research was conducted in the absence of any commercial or financial relationships that could be construed as a potential conflict of interest.

## Abbreviations

5xFAD, 5x familial Alzheimer’s disease (a TgAD model carrying 2 PSEN1 and 3βAPP mutations; see text); AD, Alzheimer’s disease; ASD, autism spectrum disorder; BD, bipolar disorder; mGlu5 miRNA, microRNA; PMS, Phelan–McDermid syndrome (22q13.3 deletion syndrome); SZ, schizophrenia.
